# Prediction of synonymous corrections by the BE-FF computational tool expands the targeting scope of base editing

**DOI:** 10.1093/nar/gkaa215

**Published:** 2020-04-07

**Authors:** Roy Rabinowitz, Shiran Abadi, Shiri Almog, Daniel Offen

**Affiliations:** Department of Human Molecular Genetics and Biochemistry, Sackler School of Medicine, Tel Aviv University, Israel; Felsenstein Medical Research Center, Tel Aviv University, Israel; School of Plant Sciences and Food Security, Tel Aviv University, Israel; Department of Human Molecular Genetics and Biochemistry, Sackler School of Medicine, Tel Aviv University, Israel; Felsenstein Medical Research Center, Tel Aviv University, Israel; Sagol School of Neuroscience, Tel Aviv University, Israel; Department of Human Molecular Genetics and Biochemistry, Sackler School of Medicine, Tel Aviv University, Israel; Felsenstein Medical Research Center, Tel Aviv University, Israel; Sagol School of Neuroscience, Tel Aviv University, Israel

## Abstract

Base editing is a genome-editing approach that employs the CRISPR/Cas system to precisely install point mutations within the genome. A deaminase enzyme is fused to a deactivated Cas and enables transition conversions. The diversified repertoire of base editors provides a wide range of base editing possibilities. However, existing base editors cannot induce transversion substitutions and activate only within a specified region relative to the binding site, thus, they cannot precisely correct every point mutation. Here, we present BE-FF (Base Editors Functional Finder), a novel computational tool that identifies suitable base editors to correct the translated sequence erred by a point mutation. When a precise correction is impossible, BE-FF aims to mutate bystander nucleotides in order to induce synonymous corrections that will correct the coding sequence. To measure BE-FF practicality, we analysed a database of human pathogenic point mutations. Out of the transition mutations, 60.9% coding sequences could be corrected. Notably, 19.4% of the feasible corrections were not achieved by precise corrections but only by synonymous corrections. Moreover, 298 cases of transversion-derived pathogenic mutations were detected to be potentially repairable by base editing via synonymous corrections, although base editing is considered impractical for such mutations.

## INTRODUCTION

Base editors (BEs) allow programmable genome editing in terms of a single nucleotide transition; purine to purine and pyrimidine to pyrimidine (A↔G and C↔T, respectively) (1,2). The base editing technology employs the clustered regulatory interspaced short palindromic repeats (CRISPR)/Cas system to deliver a deaminase protein to precise genomic loci, as directed by the guide-RNA (gRNA) (3,4). The first BE (BE1) was introduced by Komor *et al.* (1). This BE utilizes a cytidine deaminase enzyme fused to a catalytically deactivated Cas (dCas) (1), a Cas protein that contains mutations within its RuvC and HNH endonuclease domains (D10A and H840A) leading to the inability of the Cas protein to perform DNA cleavage. While the dCas protein lacks its endonuclease ability, it retains the competence to navigate through the genomic DNA to the designated locus (5). Many more variants have been devised since then (Table 1) and can be categorized to two main types: cytosine BEs (CBEs) which convert C to T and adenine BEs (ABEs) that convert A to G. The conversion by CBEs occurs via deamination of cytidine, yielding uridine that acts as thymidine in base pairing (1). ABEs utilize an adenosine deaminase enzyme to perform adenosine deamination, resulting in an inosine. During translation inosine acts as guanosine (6), hence the activity of ABE yields an A to G transition (2). By targeting the complementary strand, it is possible to indirectly convert G to A by CBE and T to C by ABE. Taken together, CBEs and ABEs are capable of performing all combinations of transition substitutions. While point mutations account for 58% of disease-causing genetic variants in humans, transition substitutions comprise 61% of the pathogenic point mutations (7).

**Table 1.** BEs repository

Base editor	Substitution	Major activity site (distance from PAM)	Minor activity site (distance from PAM)	PAM	Ref. #
BE1, BE2, BE3, HF-BE3, BE4(max), BE4-Gam	C to T	13–17	10–12, 18–19	NGG	(1,23–25)
YE1-BE3	C to T	14–16	17	NGG	(26)
YEE-BE3	C to T	15	16	NGG	(26)
VQR-BE3	C to T	10–17		NGAN	(26)
VRER-BE3	C to T	11–18		NGCG	(26)
SaBE3, SaBE4, SaBE4-Gam (21nt gRNA)	C to T	10–19		NNGRRT	(24,26)
Sa(KKH)-BE3 (21nt gRNA)	C to T	10–19		NNNRRT	(26)
Cas12a-BE	C to T	10–12 downstream	8–9, 13 downstream	TTTV	(27)
Target-AID	C to T	17–19	13–16	NGG	(28)
Target-AID-NG	C to T	17–19	13–16	NG	(29)
xBE3	C to T	13–17	10–12, 18–19	NG	(30)
eA3A-BE3	C to T when C comes after T	13–17	10–12, 18–19	NGG	(31)
BE-PLUS	C to T	7–17	5–6	NGG	(32)
CP-CBEmax variants	C to T	12–17	10–11* may exhibit editing upstream to the protospacer	NGG	(33,34)
evoAPOBEC1-BE4max	C to T	13–18	19–20, 9–12	NGG	(35)
evoFERNY-BE4max	C to T	13–18	19–20	NGG	(35)
evoCDA1-BE4max	C to T	9–20	7–8* may exhibit editing upstream to the protospacer	NGG	(35)
ABE 7.9	A to G	13–16	12, 17	NGG	(2)
ABE 7.10	A to G	14–17	13	NGG	(2)
ABE 7.10*	A to G	13–17	12,18–19	NGG	(36)
xABE, NG-ABEmax	A to G	14–17	13	NG	(30,34)
ABESa (21nt gRNA)	A to G	10–16		NNGRRT	(37)
Sa(KKH)-ABE (21nt gRNA)	A to G	10–16		NGA	(37,38)
VRER-ABE	A to G	15–17	13–14	NGCG	(37)
VQR-ABE	A to G	15–17	13–14	NNNRRT	(37,38)
CP-ABEmax variants	A to G	14–17	7–13	NGG	(33,34)

Notably, contrary to other CRISPR mediated gene editing methods, base editing does not involve DNA double-strand breaks (DSBs); thus, conferring a higher degree of safety as DSBs may result in error-prone mutagenic repair pathways (alternative end joining and single-strand annealing) (8), p53 activation (9,10), large deletions and rearrangements (11), integration of foreign genomes at the target site (12) and more. Furthermore, compared to the homology directed repair (HDR) pathway that is thought as the only precise resolution amongst DSB repair pathways, base editing is both more efficient (1) and allows editing of post-mitotic cells that are unable to undergo DSB-mediated HDR (13,14). A diverse toolbox of CBEs and ABEs is essential for developing treatments based on base editing for disease-associated point mutations. Along with the discovery of natural Cas proteins and development of synthetic variants, the BEs toolbox expands with novel CBEs and ABEs. A pivotal consideration in gRNA design in general and base editing in particular, is the protospacer adjacent motif (PAM) limitation. The PAM is a short sequence within the target DNA that has an essential role in the binding of the Cas protein. The motif must be flanking the target sequence as directed by the gRNA, downstream or upstream according to the Cas type (type II and type V, respectively) in order to induce DNA cleavage by the Cas protein (15). As the PAM determines the binding site of the Cas protein to the DNA, it dictates the activity window region of the BE. Therefore, targeting a particular nucleotide is narrowed by the presence of a PAM in a precise distance from the activity window as determined by the BE. Each BE has a major activity window, where base editing occurs most efficiently, and minor activity window(s) in which the BE exhibits some degree of editing in significantly lower rates. Within the major activity window, all the target nucleotides (C or A) are prone to undergo base editing. Consequently, if a target nucleotide is flanked by the same nucleotide, both will be edited and an unintended mutation may be introduced to the DNA (bystander base editing), instead of correction of the gene. In some cases, bystander base editing leads to a synonymous mutation compared to the intended sequence and may be accepted as a successful base editing outcome (e.g. ACTCTA [Thr,Leu] to ATTTTA [Ile, Leu] where threonine is the variant and isoleucine is the reference amino acid). A BE is comprised of Cas and deaminase enzymes fused together by a linker. Thus, each BE has its own unique features: PAM compatibility, gRNA length, orientation relative to the PAM, affinity to the target sequence, target nucleotide (C or A), efficiency, activity window width and its distance from PAM, off-targets, protein size and more.

Since single-nucleotide variants (SNVs) naturally vary in genetic context, a diverse range of BEs is essential to precisely adjust at least one to a given SNV. In base editing experimental design, one should consider the properties of the available BEs alongside their basic fit to perform transition of the target nucleotide. Due to the large selection of BEs (Table 1) and the complexity of identifying proper BEs to a target site, the necessity of a computational tool arises. gRNA design and off-targets prediction tools are available for general purposes such as gene-knockouts (16–19). However, such tools use reference genomes as a template, while point mutations and patient-derived cells differ from the reference genome and therefore such tools are not suitable for designing base editing experiments for treating point mutations. Moreover, such tools are not customized for base editing and thus, do not take under consideration the activity window of BEs and the produced coding sequence. Existing tools that are base editing oriented, do not match suitable BEs for specific SNVs (20,21), or lack the possibility to examine the translation outcome of the edited sequence (22). To magnify the potential of base editing in treating as many cases as possible, the utilization of multiple Cas varieties and the ability to translate DNA sequences and compare the editing outcome are needed. To that end, we developed BE-FF, a tool that receives SNVs data, analyzes the reference and variant sequences and their translated outcomes and matches the suitable BEs out of all available BEs. To assess the potential of base editing as a therapeutic approach for genetic diseases, we demonstrate the efficiency of BE-FF on a dataset of human pathogenic and likely-pathogenic SNVs. Furthermore, we established the BE-FF DB, a comprehensive database that includes pathogenic SNVs that can be edited via base editing.

## RESULTS

### A database of human pathogenic point mutations and their applicable BEs

**Figure 1.**
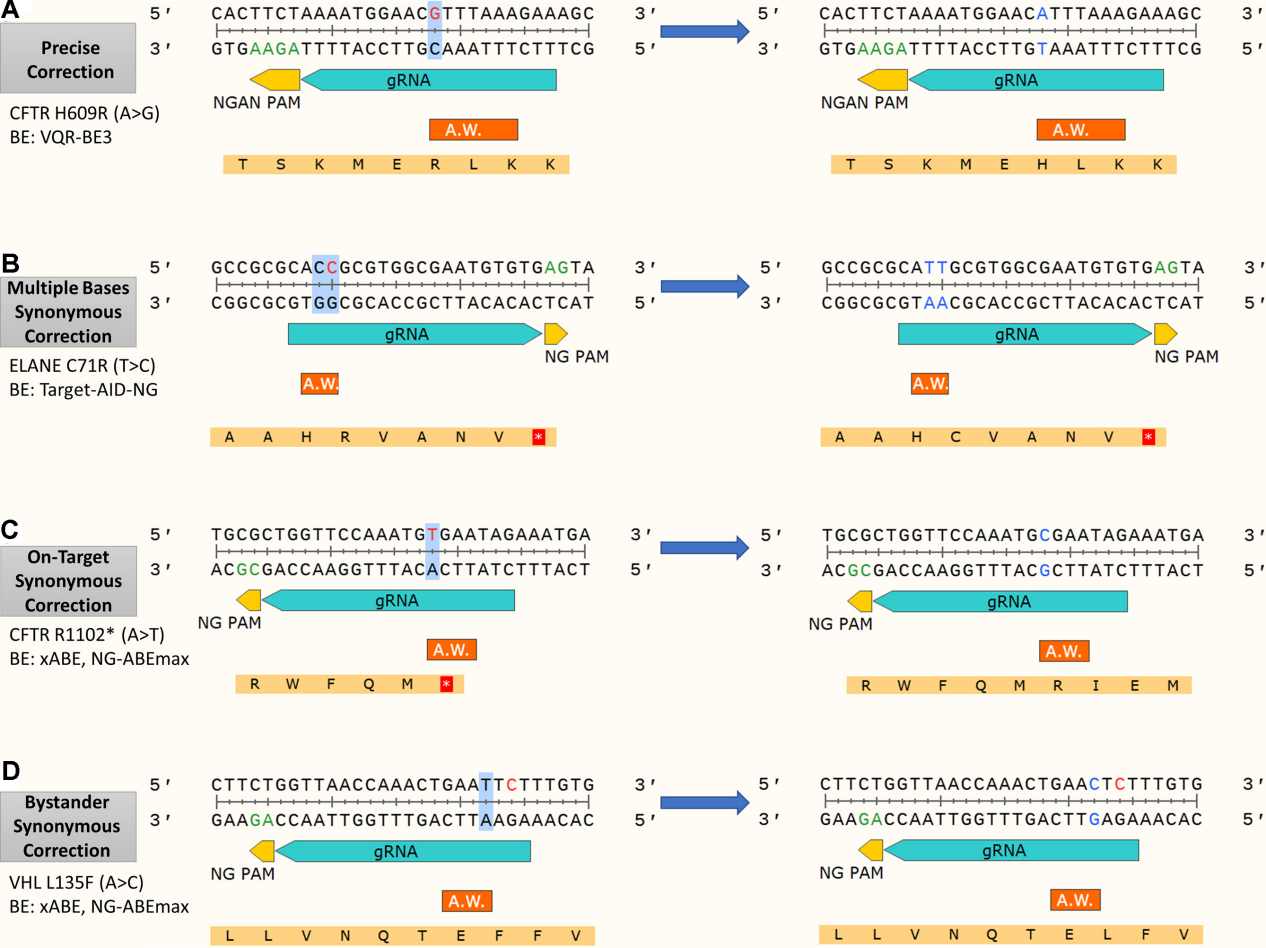
Base editing correction scenarios. The gRNA and PAM sequences appear in bright blue and yellow, respectively. The major activity window of the base editor is shown as A.W. in orange. The left sequences represent the pathogenic SNV (red) sequences and the right sequences represent the simulated base-edited (blue) sequences. The target nucleotides within the activity window are marked with blue background. (**A**) Precise correction: a transition mutation precisely repaired by VQR-BE3. (B–D) Synonymous correction scenarios. The resulting DNA sequence does not match the reference allele; however, the translated protein sequence matches. (**B**) Multiple bases synonymous correction: in addition to the target nucleotide, a bystander nucleotide lies within the activity window and undergoes base editing. (**C**) On-target synonymous correction: the variant nucleotide (T) is not restored to the reference nucleotide (A), but to another nucleotide (C). The resulted codon, however, is encoded to the reference AA. (**D**) Bystander synonymous correction: the target nucleotide remains intact while a bystander editing restores the reference protein sequence.

First, we sought to assess the potential of base editing to treat human pathogenic point mutations. To that end, we assembled a large collection of pathogenic SNVs and identified four possible scenarios of successful base editing: (i) Precise correction in which the resulting edited DNA sequence resembles the desired reference sequence (Figure 1A). (ii) Multiple bases synonymous correction: editing of the erroneous nucleotide together with bystander nucleotides that yields a correction of the target nucleotide and synonymous mutations of bystander nucleotides (Figure 1B). (iii) On-target synonymous correction is the case in which the target nucleotide is converted into a different nucleotide other than the reference one, though this conversion rescues the protein sequence, i.e. yields a synonymous codon (Figure 1C). (iv) Base editing of a bystander nucleotide within the codon of the pathogenic point mutation (bystander synonymous correction) rescues the protein sequence without editing the pathogenic SNV. The latter is mostly advantageous for transversion point mutations. While BEs are unable to reverse the DNA sequence of transversion mutations to match the reference sequence, editing a bystander nucleotide may result in a proper amino acids (AA) substitution that matches the reference protein sequence (Figure 1D). While the first two scenarios enable the correction of transition mutations, the last two scenarios also allow for the correction of transversion mutations as they exploit codon degeneracy of several AAs.

**Figure 2.**
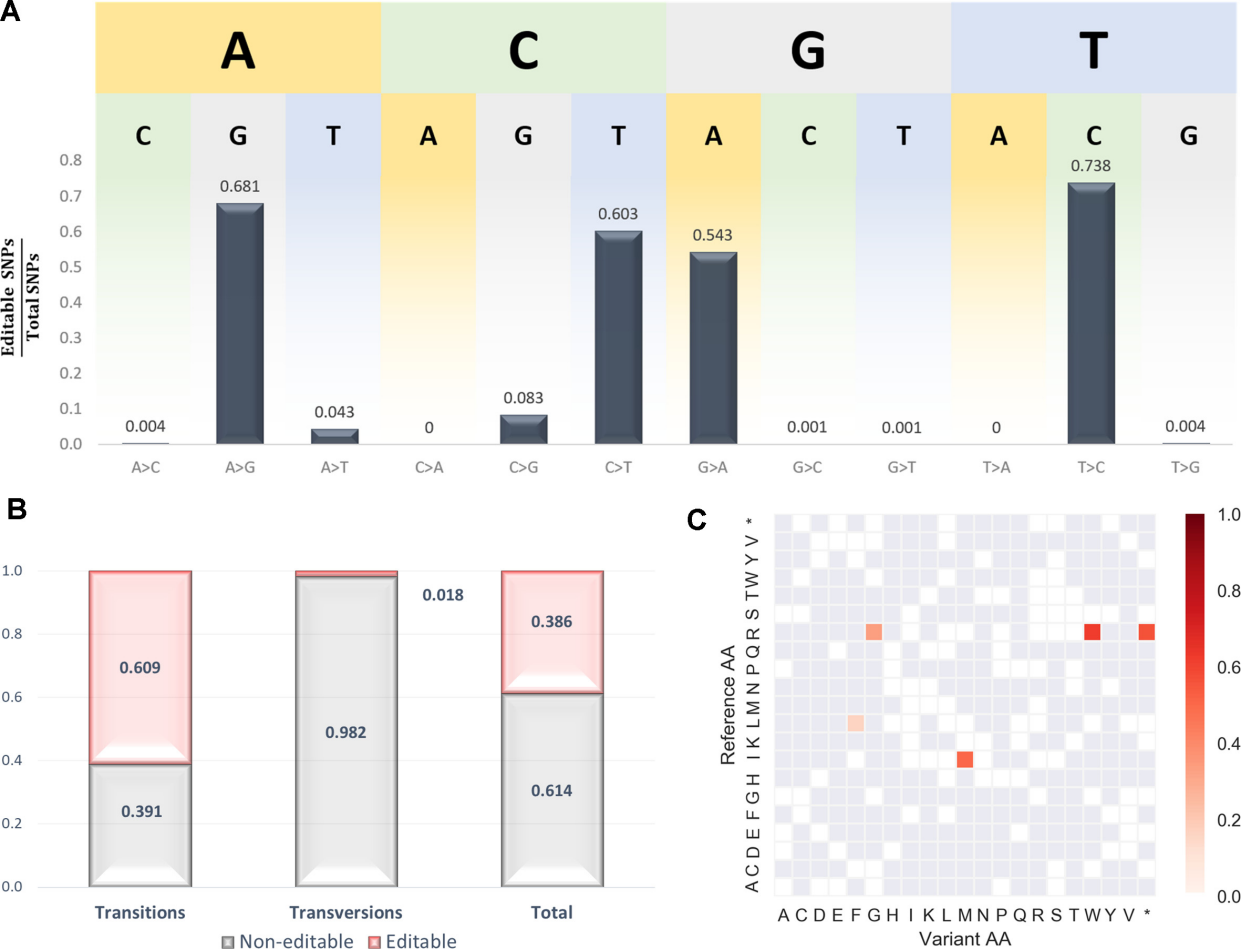
Human pathogenic mutations repaired by base editing. (**A**) Ratios of editable SNVs out of total SNVs for all substitution combinations. (**B**) Ratios of editable and non-editable SNVs for transition mutations (60.9% and 39.1%, respectively) and transversion mutations (1.8% and 98.2%, respectively). In total, 38.6% of the tested pathogenic SNVs were repairable. (**C**) Heatmap representation of the frequency of repaired transversion-derived AA substitutions (y axis - reference AAs, x axis – variant AAs). Five AA substitutions were repairable: I>M, L>F, R>G, R>W and R>* (52%, 16%, 33%, 63% and 57% of the total of the transversion-derived mutations for each, respectively).

SNVs data was obtained from NCBI’s dbSNP (https://www.ncbi.nlm.nih.gov/snp, SNVs with either pathogenic or likely-pathogenic clinical significance). The DNA sequences were fetched from Genome Browser (http://genome.ucsc.edu) and the associated phenotypes from ClinVar (https://www.ncbi.nlm.nih.gov/clinvar). Accessions of insertion, deletion or multiple nucleotide variants were excluded due to the incompatibility to correct them by a single base editing. SNVs within the mitochondrial chromosome were excluded due to the mitochondrial distinct genetic code (39). The SNVs dataset on which we performed the analysis contained 43,504 SNVs; 27,098 transitions and 16,406 transversions (supplementary file 1). In theory, any transition mutation could be corrected given that it is positioned within the major activity window of a suitable BE. Indeed, we found that 60.9% of the transitions can be repaired (Figure 2A and B). Notably, 19.4% of them could not be precisely reversed but may be corrected only by synonymous corrections (Supplementary Figure S1). Even though no existing BE can repair a transversion SNV, we detected 298 transversion-derived pathogenic point mutations (1.8% of the total transversion mutations) for which the resulting AA could be corrected, i.e. by inducing a transition editing in the SNV site or a bystander site to generate a synonymous codon (Figure 2B and supplementary file 2).

We further examined which transversion-derived AA substitutions could be corrected via base editing. According to our analysis, in case that the following transversion-derived AA substitutions occurred: I>M, L>F, R>G, R>W and R>*, a suitable BE could be found for its correction in 52%, 16%, 33%, 63% and 57% of their occurrences, respectively (Figure 2C). As expected, the majority of possible synonymous correction of transversions are those of AAs that are encoded by six codons (leucine, L and arginine, R). The third position of the isoleucine (I) codons is considered the solely threefold degenerate site, meaning that a substitution in that position to two out of three alternative nucleotides results in no change of the AA. Only a change of H (A/C/U) to G would result in an I to M missense mutation. Therefore, mutations of Isoleucine to Methionine (M) caused by transversions (T to G or C to G), could be reversed by CBEs (G to A). Serine (S), although encoded by six codons as well, could not be recovered by base editing due to the difference between its two sets of codons that do not allow the codon flexibility of leucine and arginine (UCN and AGY; where N complies to A/C/G/U thus generating a synonymous mutation in the flexible site *a-priori* and Y complies to C/U thereby only a transversion substitution could have corrected this site).

### BE-FF: base editors functional finder – a web tool that identifies BEs to correct SNVs

We established a web tool that receives SNV data and matches suitable BEs to correct the variation. The sequence and the base variant may be given manually by the user, fetched according to an rsID (accession ID in dbSNP), fetched according to genomic coordinates of 56 genomes or uploaded in a batch file. Together with the flanking regions of the SNV in the DNA sequence, the reading frame of the sequence is utilized to translate the sequence. All 26 BEs (Table 1) are available and examined to match the query. BE-FF supports user defined BE properties to allow researchers the utilization of novel or unpublished BEs. Additional BEs will be optional upon their publication. The tool does not limit the repertoire of BEs according to the base substitution. Thus, for any given SNV, an attempt to match any of the BEs is made to detect ones that perform precise correction as well as synonymous correction. The reverse-complement sequences are also considered for correction of the coding sequence. While a precise correction requires a full match of both the DNA and AA sequences, synonymous corrections are considered positive when only the AA sequences match and the DNA sequences do not. For clarity, the output is divided into two parts, precise corrections and synonymous corrections. For NGG-based BEs, off-targets assessment is offered via CRISTA (16), a third-party tool.

### Comparison to available tools

We compared BE-FF to four available base-editing design tools and report the differences in a comparative table (Table 2). Notably, BE-Designer, Benchling and beditor share a similar purpose of demonstrating the editing outcome of a user-defined base editor on a given point mutation. In contrast, BE-FF finds the suitable base editors to revert the variation of a given point mutation. BEable-GPS presents a similar utility; however, it does not consider the translated outcome of the edited sequence, therefore, if the purpose is to correct the coding sequence, it disregards possible synonymous corrections. Moreover, BEable-GPS only supports CBEs, while ABEs are excluded from its scope.

**Table 2.** Base editing tools comparative table

Tool	BE-FF	BE-Designer (20)	beditor (21)	Benchling (https://www.benchling.com/)	BEable-GPS (22)
**BE varieties^a^**	17 CBEs and 9 ABEs	Limited (3 CBEs, 1 ABE)	12 CBEs and 8 ABEs	Limited (CBEs only)	Limited (only CBEs)
**Customized user defined BE support**	Support customized deaminase type, PAM, activity window and gRNA orientation	Support customized deaminase type and activity window. Limited to predefined PAMs and their gRNA orientations	Support customized deaminase type as well as hypothetical BEs, PAM, activity window and gRNA orientation	Support customized PAMs. Customized activity window or deaminase not supported	Support customized PAM, activity window, gRNA orientation and length. Limited to CBEs only
**Identify suitable BEs to correct specific** **point mutations**	V	X	X	X	V (limited to CBEs)
**Translate editing outcome and detect synonymous corrections**	V	V	X	V	X
**Identify the BEs for correcting the outcome of a transversion mutation**	V	X	X	X	X
**User interface**	Webserver	Webserver	GUI or command line (requires installation). Limited OS support	Integrated as a feature on the Benchling web interface	Webserver
**Support multiple SNVs analysis**	V	V	V	X	X
**Off-targets assessment**	V (limited to NGG-based BEs)	V	V	V (limited to NGG-based CBEs)	X
**Targeting approach**	• Detects suitable BEs to reverse a given SNV. • Can be utilized for designing base editing-mediated therapeutics, or generating point mutations	Shows the predicted base-editing outcome for a given sequence by a user-defined BE	Shows the predicted base editing outcome for a given sequence by a user-defined BE	Shows the potential gRNAs for a user-defined DNA region within a sequence file and presents the editing outcome for the pre-defined BE.	Target region can be specified. Shows the base editing outcome of selected BEs
**Input format**	• Fetch by SNP ID • Fetch by genomic coordinates of diverse genomes • Multiple SNVs file • Standard input by user	• Multiple SNVs file • Standard input by user	Multiple SNVs file. Requires several parameters including genome, coordinates, transcript id, ref and var values for DNA and AA	DNA sequence file	Standard input by user

^a^Base editors with the same properties are counted as a single base editor. e.g. BE1, BE2, BE3, HF-BE3, BE4(max) and BE4-Gam share the same parameters and therefore considered as the same BE

## DISCUSSION

In this study, we report the feasibility of utilizing base editing to correct coding sequences that include point mutations that could not be precisely repaired. Even though several tools for base-editing design exist, BE-FF is the first to suggest BEs for the correction of the coding sequence rather than exact point-mutation. By considering mutations that induce synonymous corrections, BE-FF expands the targeting scope of base editing and in particular, allows to correct transversion-derived mutations. Our analysis demonstrates that the following transversion-derived substitutions: I>M, L>F, R>G, R>W and R>*, may sometimes be repairable via base editing and encourages considering base editing as an ideal approach for correcting such mutations. Among the repairable transversion mutations there are SNVs associated with varied conditions including cystic fibrosis, deafness, Fanconi anemia and more, emphasizing the significance of BE-FF in base editing gRNA design. We utilize the properties of 26 base editors (17 CBEs and 9 ABEs) that vary in their Cas proteins, deaminase enzymes and linkers and therefore provide a broad toolbox to perform base editing. The development of novel BEs contributes to the expansion of the base editing toolbox and advances base editing towards future therapeutics and research applications. We compared BE-FF to existing tools and found it to be valuable due to its compatibility with both CBEs and ABEs, the ability of translation and comparing the translated sequences to identify synonymous corrections, vast repertoire of BEs and a simple web-based user interface. BE-FF is ideal for finding base editing solutions to repair specific point mutations. A recent study by Anzalone *et al.* reports a novel method termed *prime editing* to make any type of edit (insertion, deletion, transition and transversion) by the CRISPR/Cas system (40). However, prime editing is more complex and requires additional refinements compared to base editing. Hence, base editing is considered favorable when possible. BE-FF is currently unable to provide off-targets assessments for BEs with a PAM other than NGG, and therefore, such off-targets assessment using complementary tools (e.g. CRISTA (16), Cas-OFFinder (41), CCTop (19) and others) is suggested. For researchers intending to make use of the BE-FF database, in case the mutation of interest is missing, it is suggested to check whether it appears on the full dataset we used. Otherwise, it is recommended to use the web tool to analyze the variation of interest. Moreover, BE-FF detects possible minor editing according to the defined positions of the minor activity window. In such cases, an indication will appear stating that minor editing may occur. It is recommended to compare the actual experimental outcomes of the candidate BEs to identify the most suitable BE.

## DATA AVAILABILITY

The BE-FF web tool is freely available at: https://www.danioffenlab.com/be-ff.

The code is available at: https://github.com/RoyRabinowitz/BE-FF.

## IMPLEMENTATION

BE-FF is web-based and does not require installation or specific specifications.

## Supplementary Material

gkaa215_Supplemental_Files
