# Supplementary material for: Prediction of synonymous corrections by the BE-FF computational tool expands the targeting scope of base editing
Source: Nucleic Acids Res. 2020 Apr 7;48(W1):W340–7. doi: 10.1093/nar/gkaa215 (PMC7319459; doi:10.1093/nar/gkaa215)
Supplement: gkaa215_Supplemental_Files [file gkaa215_supplemental_files.zip › Supplementary Figure1.docx]

**BE-FF: Base Editors Functional Finder tool - Supplementary File**

**Supplementary figure 1**


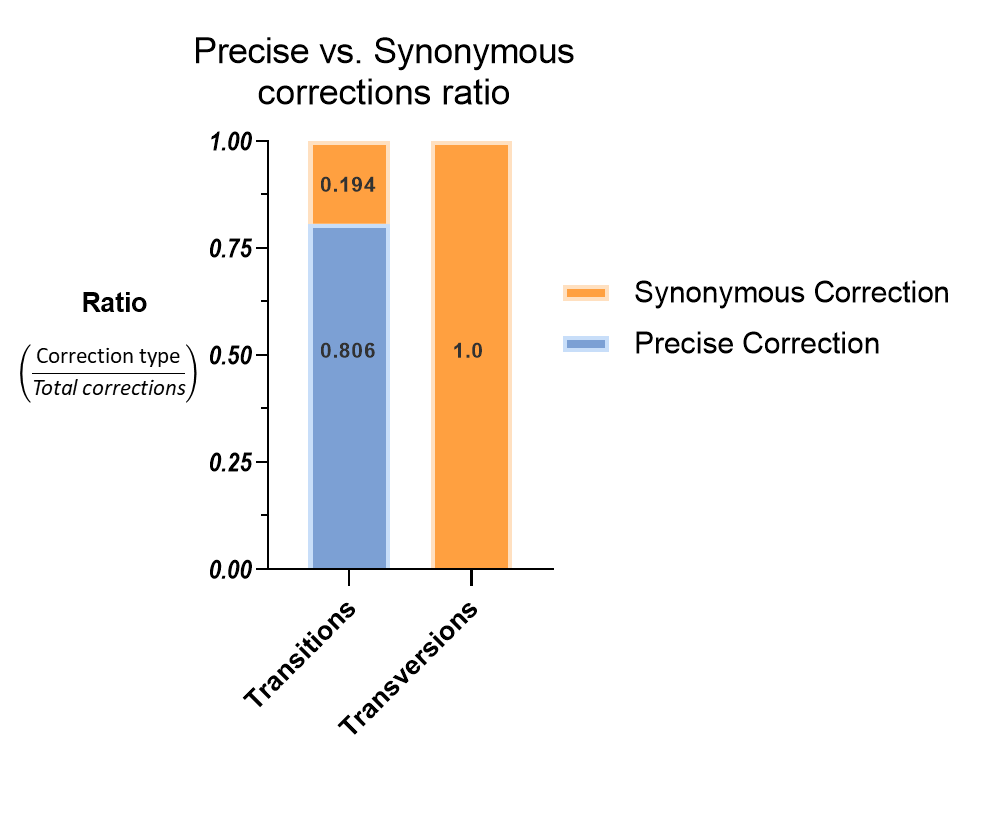


**Figure S1: Precise and synonymous correction ratios**. The SNVs that could be repaired by precise correction only, or by both precise and synonymous corrections were summed and divided by the total repairable SNVs. SNVs that could be repaired only by synonymous correction were summed and divided by the total repairable SNVs.
